# *In-utero* epigenetic factors are associated with early-onset myopia in young children

**DOI:** 10.1371/journal.pone.0214791

**Published:** 2019-05-17

**Authors:** Wei Jie Seow, Cheryl S. Ngo, Hong Pan, Veluchamy Amutha Barathi, Stuart W. Tompson, Kristina N. Whisenhunt, Eranga Vithana, Yap-Seng Chong, Suh-Hang H. Juo, Pirro Hysi, Terri L. Young, Neerja Karnani, Seang Mei Saw

**Affiliations:** 1 Saw Swee Hock School of Public Health, National University of Singapore and National University Health System, Singapore; 2 Department of Medicine, Yong Loo Lin School of Medicine, National University of Singapore and National University Health System, Singapore; 3 Department of Ophthalmology, National University Health System, Singapore; 4 Singapore Institute for Clinical Sciences (SICS), A*STAR, Brenner Centre for Molecular Medicine, Singapore; 5 Singapore Eye Research Institute, Singapore; 6 The Ophthalmology and Visual Sciences Academic Clinical Program, DUKE-NUS Graduate Medical School, Singapore; 7 Department of Ophthalmology, Yong Loo Lin School of Medicine, National University of Singapore, Singapore; 8 Department of Ophthalmology and Visual Sciences, University of Wisconsin–Madison, Madison, Wisconsin, United States of America; 9 Department of Obstetrics and Gynaecology, National University of Singapore, Singapore, Singapore; 10 Institute of New Drug Development, Center for Myopia and Eye diseases, China Medical University and China Medical University Hospital, Taichung, Taiwan; 11 Department of Twin Research and Genetic Epidemiology, King's College London, London, United Kingdom; 12 Johns Hopkins School of Public Health, Baltimore, Maryland, United States; University of Rochester, UNITED STATES

## Abstract

**Objectives:**

To assess whether epigenetic mechanisms affecting gene expression may be involved in the pathogenesis of early-onset myopia, we performed genome-wide DNA methylation analyses of umbilical cord tissues, and assessed any associations between CpG site-specific methylation and the development of the disorder when the children were 3 years old.

**Methods:**

Genome-wide DNA methylation profiling of umbilical cord samples from 519 Singaporean infants involved in a prospective birth cohort ‘Growing Up in Singapore Towards healthy Outcomes’ (GUSTO) was performed using the Illumina Infinium HumanMethylation450K chip microarray. Multivariable logistic regression models were used to assess any associations between site-specific CpG methylation of umbilical cord tissue at birth and myopia risk in 3 year old children, adjusting for potential confounders. Gene expression of genes located near CpG sites that demonstrated statistically significant associations were measured in relevant ocular tissues using human and mouse fetal and adult eye samples.

**Results:**

We identified statistically significant associations between DNA methylation levels at five CpG sites and early-onset myopia risk after correcting for multiple comparisons using a false discovery rate of 5%. Two statistically significant CpG sites were identified in intergenic regions: 8p23(p = 1.70×10^−7^) and 12q23.2(p = 2.53×10^−7^). The remaining 3 statistically significant CpG sites were identified within the following genes: *FGB* (4q28, p = 3.60×10^−7^), *PQLC1* (18q23, p = 8.9×10^−7^) and *KRT12* (17q21.2, p = 1.2×10^−6^). Both *PQLC1* and *KRT12* were found to be significantly expressed in fetal and adult cornea and sclera tissues in both human and mouse.

**Conclusions:**

We identified five CpG methylation sites that demonstrate a statistically significant association with increased risk of developing early-onset myopia. These findings suggest that variability in the neonatal cord epigenome may influence early-onset myopia risk in children. Further studies of the epigenetic influences on myopia risk in larger study populations, and the associations with adulthood myopia risk are warranted.

## Introduction

Myopia is a common eye disorder and a major public health concern due to its high prevalence especially across urban Asian regions[1, 2]. Children who develop myopia at an early age have a greater risk of developing high-grade myopia as adults[3]. Adults with high-grade myopia are at an increased risk of developing retinal detachment, myopic macular degeneration and glaucoma, all of which result in irreparable loss of vision[4–6]. Therefore, it is imperative to better understand the etiology of myopia, especially the early-onset forms.

Genetics, as well as environmental factors such as near work and outdoor time have been shown to be risk factors for myopia[7–13]. The Consortium for Refractive Error and Myopia (CREAM) has performed genome-wide meta-analyses, studying 37,382 individuals from 27 cohorts of European ancestry, and 8,376 individuals from 5 Asian cohorts, and has identified new loci associated with refractive error risk[14]. Similarly, epigenetic mechanisms affecting gene expression may be involved in the pathogenesis of eye diseases such as myopia[15]. For instance, in a murine form deprivation model of myopia, disease development has been associated with changes to scleral DNA methylation at CpG sites in the collagen 1A1 (*COL1A1*) gene promoter and altered mRNA expression levels[16].

Examination of epigenetic markers at birth could provide novel insights into the etiology of early-onset myopia, and modification of reversible, environmental factors could influence the refractive error outcome in both childhood and adulthood. However, currently there are no epigenetic genome-wide studies of myopia risks. The aim of our study is to examine whether there are any associations between umbilical cord tissue DNA methylation sites and subsequent early-onset myopia risk among Singaporean children in the GUSTO birth cohort.

## Materials and methods

### Study population

The Growing Up in Singapore Towards healthy Outcomes (GUSTO) naturally-conceived birth cohort consists of pregnant women aged 18 years and older, who attended their first trimester routine antenatal ultrasound scan at either one of the two major maternity units at the Kadang Kerbau Women’s and Children’s Hospital (KKH) or at the National University Hospital (NUH) between June 2009 and September 2010.[17] This study enrolled mothers and their directly descended children of Chinese, Malay and Indian origin which are the 3 major ethnic groups in Singapore. Children who developed strabismus, facial nerve palsy, eye infection, developmental anomaly, eye injury or any other ocular conditions by age 3 were excluded from the study. The study was approved by the SingHealth Centralized Institutional Review Board and the National Health Group’s Domain Specific Review Board, and was conducted according to the tenets of the Declaration of Helsinki. Informed written consent was obtained from the parents or legal guardians following an in-person interview that included a detailed verbal explanation of the study.

### Eye measurements performed at 3 years of age

One drop of 0.5% proparacaine and one drop of 2.5% phenylephrine were followed by three drops of 1% cyclopentolate instilled at 5-minute intervals in order to achieve cycloplegia. Cycloplegic autorefraction was measured using a table-mounted Model RK-F1 autorefractor (Canon, Tokyo, Japan) 30 minutes after the administration of the last eye drop. Each eye’s spherical equivalent refraction (SER) was calculated as the sphere power plus half the cylinder power. Myopia was defined as a SER magnitude of at least -0.5 dioptres (D). Disease-free controls were defined as children without a myopic SER.

### DNA methylation analysis

Genomic DNA was extracted from the infant umbilical cords of GUSTO samples collected at birth, bisulfite-converted and hybridized to Infinium Human Methylation450 BeadChip microarrays (Illumina, Inc., San Diego, California, USA) as per the manufacturer’s instructions[18]. The raw signals were exported using the GenomeStudio Methylation Module (Illumina, Inc., San Diego, California, USA). Following standard protocol and quality control procedures[19], probes with less than three beads for any sample, or with signal detection p-values (calculated from the signal versus background for the individual bead intensities) > 0.01 for any sample were discarded.

Percentage methylation or β-values, were calculated as the ratio of the methylated probe intensity to the overall CpG site intensity for each CpG site, resulting in values ranging from 0 to 1. Batch effects were removed using COMBAT[20]. CpG sites that were cross-hybridizing, overlapping single-nucleotide polymorphisms (SNPs), or those where the methylation range across the study subjects was less than 10%, were excluded.

To correct for cell-type heterogeneity, hematocyte proportions were estimated for fibroblasts, B-cell and T-cell lymphocytes using the Houseman reference panel method[21, 22]. Using principal component analysis (PCA) on the cellular proportion matrix, the first two principal components were extracted as surrogates for the primary hematocyte distribution.

### Validation gene expression in human fetal and adult eye tissues

Human fetal and adult gene expression was measured in the retina, retinal pigment epithelium (RPE), choroid, sclera, optic nerve and cornea as described in Young TL, et al[23]. Briefly, 8 fetal eyes at 12-weeks and 6 fetal eyes at 24-weeks gestational age were obtained from Advanced Biosciences Resources (Alameda, California, USA). In addition, 6 adult eyes were obtained from the North Carolina Eye Bank (Winston–Salem, North Carolina, USA). RNA was extracted using a mirVanaTM total RNA extraction kit (Ambion, Austin, Texas, USA) following the manufacturer's protocol. The RNA samples were amplified using an Illumina Total Prep kit (Ambion, Austin, Texas, USA) and hybridized to Illumina HumanHT-12 v4 Expression BeadChips (San Diego, California, USA). Twelve tissue samples were processed on each chip. Microarray data background noise was subtracted from the intensity values using Illumina’s GenomeStudio software, exported and log2 transformed. Sample outliers were determined by principle component analyses using Hoteling's T2 test[24] at a 95% confidence interval and removed from further analyses. Data intensities were normalized by Quantile normalization followed by Multichip Averaging to reduce chip effects[25]. In instances where a gene had multiple probes on the array, the corresponding p-values were combined using Fisher’s method (Fisher's combined probability test) and the signal intensities were averaged.

### Animal experimentation

C57BL/6J wild type (WT) mice were purchased from National University of Singapore (Singapore). Animals were housed on a 12 hour light/ 12 hour dark cycle with food and water provided ad libitum. SingHealth Institutional Animal Care and Use Committee (IACUC; AALAC accredited) has approved the study and animal procedures. The animal procedures performed in this study were in accordance with the Guide for the Care and Use of Laboratory Animals. All aspects of the study were in accordance with the Association for Research in Vision and Ophthalmology (ARVO) recommendations for animal experimentation.

### Murine myopia model

A −15 diopter spectacle lens [PMMA Contact Lens (Lenspec, Singapore) in Grey Tint, 8.5 mm diameter, 8 mm base curve, refractive index: 1.43, axial thickness: 0.5 mm] was placed over the right eye on post-natal day 10 before eye opening by gluing to an annulus of matching Velcro tape surrounding the periorbital skin. The spectacle lenses were cleaned daily in dim light. The left eyes were uncovered and served as controls. All optical interventions were removed on postnatal day 52 [26–28].

### Ocular biometry assessment

The refractive error of each eye was measured weekly using an automated infrared photorefractor (Image Source, Kirkland, WA). By using optical low coherence interferometer (OLCI), AC Master (Carl-Zeiss Meditec, Oberkochen, Germany), the biometry of the eye was measured *in vivo* [29, 30] at days, 24, 31, 38, 45 and 52 after induction of myopia. Differences of refractive power, axial length, corneal thickness, anterior chamber depth, lens thickness and vitreous chamber depth between treated and control eyes were calculated.

### Validation gene expression in mouse eye tissues

Total RNA was isolated from single cryogenically ground WT mouse retina and sclera (n = 12 eyes from myopic, control and naïve group; conducted in 2 batches; n = 6 samples from each group from each batch) using RNeasy Mini kit (Qiagen, Germany). The concentration and quality of RNA was determined by absorbance at 260 nm and the absorbance ratio of 260/280 using the Nanodrop ND-1000 Spectrophotometer (Nanodrop Technologies, Wilmington, DE). Subsequently, the isolated RNA was reverse-transcribed into sense cDNA using a T7-N6 primer, labelled with biotin using a Genechip Whole Transcript Sense Target Labeling Assay (Affymetrix, Inc., Santa Clara, CA) and hybridized to a Mouse Gene 1.0 ST Array (Affymetrix, Inc.) using the Genechip Hybridzation Kit (Affymetrix, Inc.). The microarray chips were then stained using a Genechip Hybridzation, Wash and Stain Kit (Affymetrix, Inc.) and scanned using a Genechip Scanner 3000 7G (Affymetrix, Inc.).

### Statistical analysis

Characteristics between the myopic children and controls were compared using the Wilcoxon rank sum test for continuous variables and Fisher’s exact test for categorical variables. Multivariable linear regression models were used to assess any association between myopia onset status in 3 year old children (binary variable) and genome-wide, CpG site-specific DNA methylation of umbilical cord tissues at birth, adjusting for sex, ethnicity (Chinese, Malay and Indian), gestational age (continuous value in weeks), the first two principal components of hematocyte proportion, and bisulfite conversion plate batch effects (dummy values). We also used continuous SER and adjusted for parental smoking as well as parental myopic refractive status as a potential confounder in the sensitivity analysis. The resulting p-values were corrected for multiple testing using the Benjamini and Hochberg[31] method and were considered genome-wide significant with a false discovery rate (FDR) < 0.05. A Bonferroni-corrected p-value for the multiple testing of all 160,418 CpGs was also used as a threshold for significance; the corrected p-value was 0.05/160,418 = 3.10 × 10^−7^. Due to the high correlation between right and left eyes, we used the average SER from both eyes (Spearman rho: 0.88).

All statistical tests were two-sided and p < 0.05 was considered statistically significant. All data analysis and statistical tests were conducted in R version 3.0 and above (www.r-project.org) and STATA (StataCorp. 2009. Stata Statistical Software: Release 11. College Station, TX: StataCorp LP.).

## Results

Out of the 1,236 recruited participants, 925 children (74.8%) attended a clinical follow-up visit on the third year. Cycloplegic refraction were measured in 574 children (46.3%) respectively[32]. A total of 519 children with both umbilical cord methylation profiles and cycloplegic refraction measurements were included in this epigenome-wide association study (EWAS) study. Following the removal of CpG sites that cross-hybridized, overlapped with SNPs, or demonstrated a methylation range across the study subjects of less than 10%, the total number of CpG sites that remained for further analysis was 160,418[33, 34].

There were 29 myopic cases (SER < -0.5D) and 490 non-myopic controls (S1 Table). SER data was available for all 519 children at 3 years of age, of which 258 were males and 261 were females. There were 295 (56.8%) Chinese, 71 (26.6%) Malay and 42 (16.6%) Indian participants. The mean gestational age was 38.1 weeks (standard deviation (SD) = 1.4). The mean SER for all subjects was 0.90D (SD = 1.01D) with a range of -7.25D to 4.01D.

**Fig 1:**
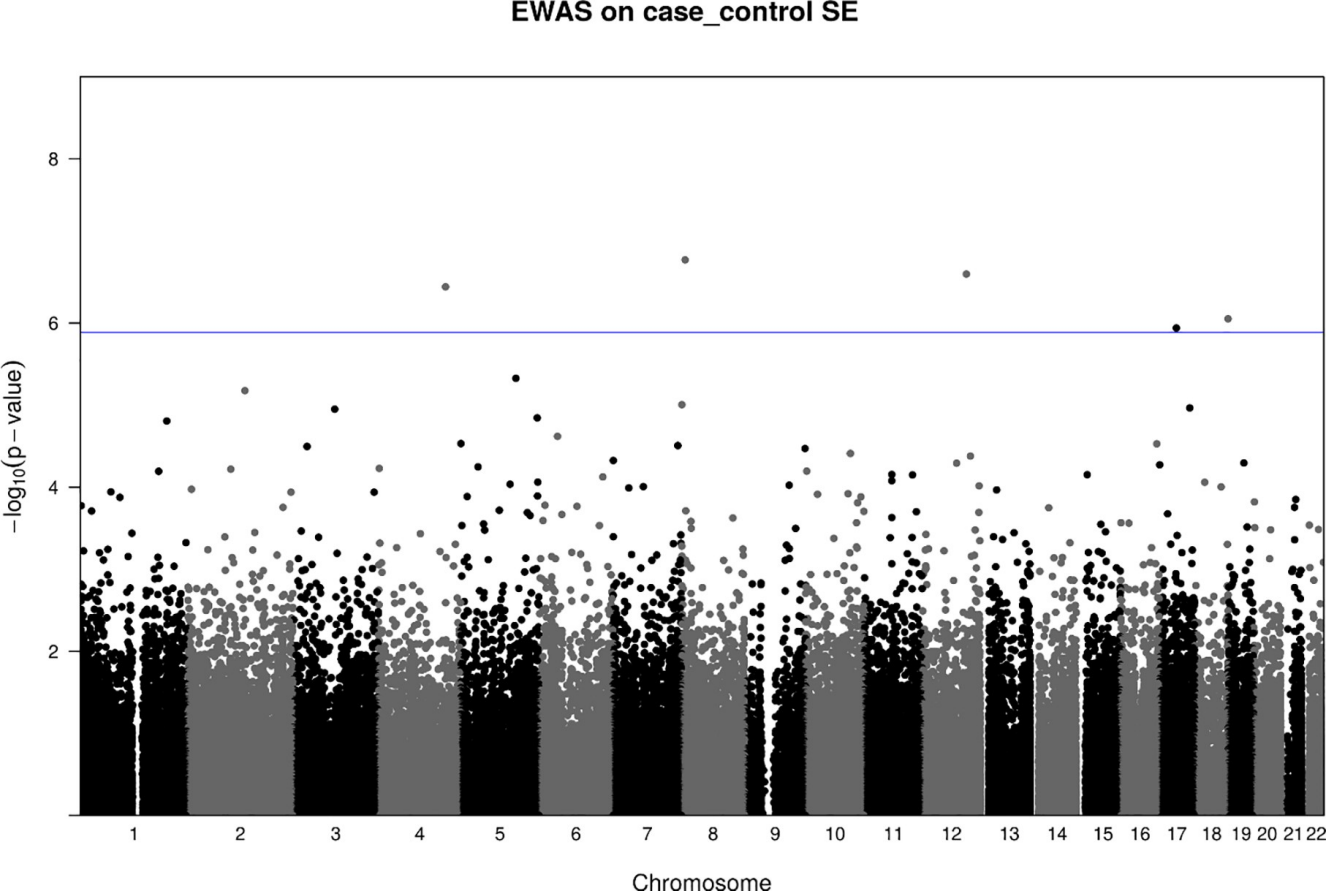
Manhattan plot of the EWAS results on umbilical cord methylation profiles. Each point represents a CpG site (n = 160,419) with the chromosomal position along the x-axis and the negative logarithm of the associated p value on the y-axis. The solid blue horizontal line indicates FDR at 5%, which was 1.30 × 10^−6^ in this study. The results were from linear regression of methylation on case-control groups adjusted by covariates including child gender, ethnicity, cell types, bisulfite conversion batch and gestational age. Data showed there were 5 CpGs above the line of FDR corrected p-value after adjusting by covariates.

**Fig 2:**
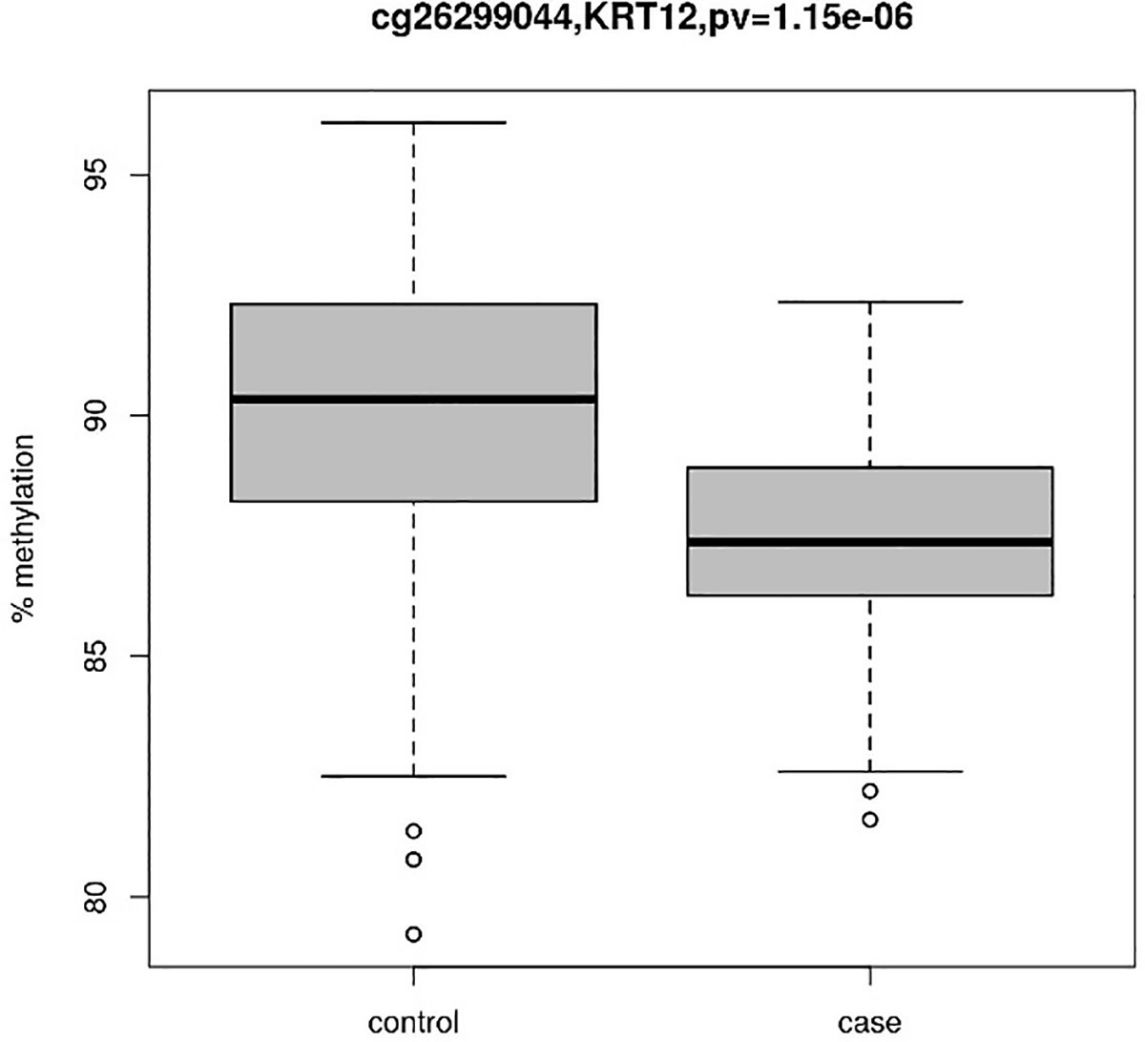
Methylation profiles of the significant CpG sites for myopic cases and controls.

After adjusting for sex, ethnicity, gestational age, cellular composition and bisulfite conversion plate batch effects, we found five CpG probes (cg21880079, cg14066632, cg03155767, cg17154092, cg26299044) whose mean methylation levels were statistically significantly different between cases and controls (p-values < 3.10×10^−7^). All five probes passed 5% FDR, of which three CpG probes passed Bonferroni correction (Table 1, Fig 1). The distribution of observed p-values for each CpG site followed the expected distribution for most of the CpGs (S1 Fig). All five detected CpG probes were significantly hypomethylated among the 3-year old myopia cases compared to controls (Fig 2). Using continuous SER measures and adjusting for parental myopic refractive status as well as parental smoking status did not change the results (S2–S4 Tables).

**Table 1:** Significant CpGs that are different between myopic cases and controls at a 5% FDR.

CpG	Chr	Estimate (95% CI)_a_	P-value_b_	Nearest gene	Gene location	FDR
cg21880079	8p23	-3.06 (-4.20, -1.93)	1.70 × 10–7	-	Intergenic	0.019
cg14066632	12q23.2	-3.54 (-4.87, -2.21)	2.53 × 10–7	*ARL1*	Intergenic	0.019
cg03155767	4q31.3	-2.40 (-3.32, -1.49)	3.62 × 10–7	*FGB*	3' UTR (exon 8 of 8)	0.019
cg17154092	18q23	-4.49 (-6.27, -2.72)	8.88 × 10–7	*PQLC1*	intron (intron 3 of 3)	0.036
cg26299044	17q21.2	-2.81 (-3.93, -1.69)	1.15 × 10–6	*KRT12*	intron (intron 2 of 7)	0.037

Abbreviations: Chr, chromosome; IQR, inter-quartile range; CI, confidence interval; FDR, false discovery rate

^a^Regression coefficients (Estimate) are reported as percentage methylation change in case group.

^b^P-value was obtained from linear regression of myopia (case and control) and methylation at each CpG site, adjusted for sex, gestational age, ethnicity, bisulfite conversion batch and cellular proportions.

To assess whether the four genes identified at or near statistically significant CpG probes (*ARL1*, *FGB*, *PQLC1*, *KRT12*) were expressed in myopia-relevant ocular tissues, gene expression data from human fetal and adult ocular tissues were examined. The *ARL1* gene was expressed in adult retina (p = 1.0×10^−15^), RPE (p = 0.0037), sclera (p = 0.0004), optic nerve (p = 0.0002) and cornea tissue (p = 1.0×10^−15^), and fetal retina/RPE (p = 1.0×10^−15^), choroid (p = 1.0×10^−15^), sclera (p = 1.0×10^−15^), optic nerve (p = 1.0×10^−15^) and cornea tissue (p = 1.0×10^−15^). *KRT12* was found in adult (p = 1.0×10^−15^) and fetal cornea (p = 1.0×10^−15^) tissue. *PQLC1* gene expression was found in adult (p = 0.0037) and fetal retina/RPE (p = 1.0×10^−15^), sclera (p = 1.0×10^−15^), choroid (p = 1.0×10^−15^), optic nerve (p = 1.0×10^−15^) and cornea tissue (p = 0.0002). However, *FGB* was not found to be expressed in any human eye tissues.

Furthermore, we evaluated the four genes for expression in a myopic animal model. We found that *PQLC1* (p = 1.50×10^−1^), *KRT12* (p = 1.99×10^−1^) and *FGB* (p = 3.60×10^−7^) gave an expression signal in the mouse sclera, but not in mouse retina. However, only the *FGB* expression signal in the mouse sclera was statistically significant.

## Discussion

To our knowledge, this is the first epigenome-wide association study of early-onset myopia among children. We have identified statistically significant differential methylation at five CpG sites that associate with early-onset myopia. The genomic loci for these CpG sites are an intergenic region on 8p23, a region near the ARL1 gene on 12q23.2, and regions within the *FGR*, *PQLC1* and *KRT12* genes. Our microarray data also shows that three of these genes are expressed in human fetal eye tissues (*ARL1*, *KRT12*, and *PQLC1)*, of which two (*PQLC1* and *KRT12*) are also expressed in a mouse model of myopia, leading to the hypothesis that these CpG sites may be biologically relevant. All five CpG sites showed a reduction in methylation among myopic children versus non-myopic children, which may correlate with gene expression alterations and lead to a higher likelihood of developing myopia in early childhood.

A considerable amount of research has been conducted on the molecular determinants of myopia. GWAS studies of refractive error involving large consortia, such as the 23andMe and CREAM groups, have identified more than 100 gene loci with a high degree of concordance between studies[35]. A number of associating SNPs have been located within or near genes known to be related with eye and neuronal development and signalling, the retinal visual cycle of the retina and general eye structure. However, these loci together explain only a small percentage of the observed variation in refractive error (2.3% in a study of children)[36]. Animal models of myopia using retinal gene profiling have also identified a large number of genes that may control eye growth[37]. However, there have been very few studies investigating whether epigenetic changes may be involved in the development of myopic disease. Interestingly, a study by Zhou et al.[16] found that in a monocular form deprivation-induced mouse model of myopia, eyes with refractive error showed a higher frequency of scleral DNA methylation at the *COL1A1* promoter and a reduced level of *COL1A1* mRNA[16]. This study, which focused specifically on the *COL1A1* gene, suggested that myopia could be caused by an inhibition of scleral collagen production.

In our genome-wide association study of CpG methylation and early-onset myopia, we have identified epigenetic variation at five CpG sites at or near four gene loci that significantly associate with early-onset myopia. These genes (*ARL1*, *KRT12*, *PQLC1* and *FGB*) are known to be involved in membrane transport, as well as corneal epithelium development[38, 39]. Previous studies have reported mRNA expression of *ARL1*, *KRT12*, *PQLC1* and *FGB* genes in the brain, cortex and cerebellum^30^. Consistent with earlier findings, previously identified genes related to myopia were also located within or near genes associated with the brain, neuronal development and signalling^28^. It has previously been proposed that the development of myopic eye growth is initiated by a visually evoked signalling cascade starting from the sensory retina, passing through the RPE and choroid, and ending with altered scleral physiology, where extracellular matrix remodelling such as thinning and stretching results in the elongation of the eye^31^.

The most significant CpG site, cg21880079, which is differentially methylated between myopic cases and controls is located in an intergenic region of chromosome 8p23. Interestingly, 8p23 is a known myopia locus (MYP10) that was previously reported in two family-based association studies[40, 41] and has also been associated with high myopia in the French population[42]. The high myopia association is particularly interesting, as early-onset of myopia in children has been shown to be the most important predictor of high myopia in later childhood[3]. CpG site cg21880079 is also located within exon 3 of the long intergenic noncoding RNA (lincRNA) ENST00000524073, however its function is currently unknown.

The second CpG site, cg14066632, is located 4kb upstream of the *ARL1* gene on chromosome 12, which encodes a protein important for the normal function of the Golgi apparatus^30^. Similar to *PQLC1*, *ARL1* is also significantly expressed in adult and fetal eye tissues. Given the proximity of this CpG site to the gene, it is likely that it is located close to the promoter of *ARL1*, hence any change of methylation in this region could lead to differential regulation of the expression of *ARL1*, given that methylation may affect the binding of transcription factors in promoter regions.

The third CpG site, cg03155767, is located within the 3’ UTR of the *FGB* (Fibrinogen Beta Chain) gene on chromosome 4, which encodes for the beta component of fibrinogen. The methylation site is located in close proximity to the stop codon located within the last exon. Mutations in this gene have been linked to several disorders, including afibrinogenemia, dysfibrinogenemia and hypodysfibrinogenemia[43, 44]. *FGB* has a major function in hemostasis and could also facilitate the antibacterial immune response via both innate and T-cell mediated pathways[45, 46]. However, its ocular functions and possible contribution to the pathogenesis of myopia are unclear.

The fourth significantly associating CpG site, cg17154092, is located in an intronic region of the *PQLC1* (PQ Loop Repeat Containing 1) gene. *PQLC1* is a protein-coding gene, which maps to chromosome 18. Members of this gene family are membrane bound proteins that are likely to function as membrane transporters of cysteine, cationic amino acids, lysine and/or arginine across the lysosomal membrane in order to maintain acidic homeostasis^35^. Intriguingly, the CREAM consortium previously identified a number of genes implicated in ion transport, such as *KCNQ5* and *KCNJ2* that are involved in transporting potassium ions^36^. Furthermore, our study detected significant levels of *PQLC1* gene expression in adult and fetal eye tissues. Therefore, *PQLC1* could participate in transporting ions across ion channels in the photoreceptors associated with myopia.

The fifth, significantly hypomethylated, CpG site is cg26299044, which is located in an intronic region of the *KRT12* gene on chromosome 17. *KRT12* encodes the type I intermediate filament chain keratin 12, expressed in corneal epithelia. Mutations in this gene are known to cause corneal dystrophy. During embryonic development, *KRT12* is expressed in corneal peridermal epithelium, and increases in expression as the corneal epithelium matures during development^37^. Both detected CpG sites of *PQLC1* and *KRT12*, which were found to be significantly expressed in fetal and adult cornea and sclera tissues in both human and mouse, are located in the intron regions of these two genes. Hypomethylation of introns has been consistently shown to be correlated with higher levels of gene expression across tissues and species [47, 48]. Therefore, hypomethylation of intronic CpG sites might lead to increased gene expression of *PQLC1* and *KRT12* in the myopic cases. Scleral changes are one of the primary determinants of eye size and consequently, refractive status of the eye. Further studies can be done to investigate if the hypomethylation of these CpG sites are correlated with changes in cornea convexity and myopia.

A limitation of this study is that fetal cord cells were obtained from the umbilical cord at birth, while the cycloplegic refraction (major phenotype) was performed at 3 years of age. Tissue specific DNA methylation is an important issue in EWAS, but due to the difficulty in obtaining tissues from eyes, especially for large cohort studies, we could only use surrogate tissue. We postulate that the biological changes seen in early-onset myopia in very young children are already reflected in umbilical cord tissue at birth. However, we recognise that DNA methylation in umbilical cord tissue might not necessarily extrapolate to the tissues of interest such as the eye or the brain. However, if the objective is to identify a biomarker for disease risk, then development of such assays has clear clinical applications. Epigenetic studies on neurodevelopment have found that *in utero* DNA methylation changes can be used to predict various future outcomes in human studies[49–52]. Therefore, DNA methylation changes in the umbilical cord tissue may also predict phenotypic outcomes in the eye. We understand that there is a need to construe the results with caution to prevent over-interpretation, hence further large-scale epigenetic studies to examine how these markers progress with time and correlate with myopia, as well as how umbilical cord tissue methylation correlates with human eye methylation are warranted.

Confounding by cellular heterogeneity is a concern for DNA methylation data analyses. Cord tissue is heterogeneous in its cellular content and consists of stromal, epithelial and endothelial cells. Different cell types have distinct methylation profiles and differences between cord methylation and eye methylation profiles are not well documented. To combat the issue of cellular heterogeneity, we used cell type reference set analyses, though residual confounding effects can still persist. Availability of better cell type reference sets developed by cell fractionation of cord tissue will help alleviate this limitation in future. Further epigenetic studies at older age groups can be done to study how these markers progress with time and correlate with myopia. We studied the expression of these epigenetically modified gene loci in adults as well as fetal human eyes, however these were not myopic conditions. The small sample size resulted in a small number of cases in the case-control analyses, however our results were statistically significant after adjusting for multiple comparisons.

To our knowledge, there are no other studies associating *in-utero* epigenetic factors with early-onset myopia in young children. This work capitalizes on a unique opportunity to assess the epigenome in a well-characterized early-onset myopic cohort. Early environmental exposures such as stress, or environmental light[53], could have an effect similar to air pollution[54], cigarette smoking[55] or other environmental factor influencing the epigenome in other diseases. Another major strength of this study is the prospective study design, where methylation was measured before the development of myopia and therefore eliminating the effects of disease bias and reverse causation.

In summary, we identified an association between early-onset myopia and variation in the epigenome. Replication of these findings in other population studies as well as their longitudinal follow-up from birth to onset of myopia will help strengthen and advance these findings.

## Supporting information

S1 Table Characteristics of study population in EWAS myopia case-control study. (DOCX)

S2 Table Significant CpGs that are different between myopic cases and controls using continuous SER measures. (DOCX)

S3 Table Significant CpGs that are significant different between cases and controls, adjusting for parental myopic status. (DOCX)

S4 Table Significant CpGs that are different between myopic cases and controls at a 5% FDR, adjusting for maternal smoking status. (DOCX)

S1 Fig Q-Q plot for p-values from EWAS on umbilical cord methylation profiles from case-control comparisons. (DOCX)
