# Supplementary material for: In-utero epigenetic factors are associated with early-onset myopia in young children
Source: PLoS One. 2019 May 17;14(5):e0214791. doi: 10.1371/journal.pone.0214791 (PMC6524791; doi:10.1371/journal.pone.0214791)
Supplement: S1 Table — (DOCX) [file pone.0214791.s001.docx]

Supplementary Table 1. Characteristics of myopia case-control population in epigenome-wide association study

| Characteristics | Cases | Controls | P-value |
| --- | --- | --- | --- |
|  | 29 | 490 |  |
| Gestational age in weeks (SD) | 38.43 (1.77) | 38.83 (1.37) | 0.13 |
| Male (%) | 17 (58.6%) | 241 (49.2%) | 0.32 |
| Ethnicity  Chinese (%) | 18 (62.1%) | 277 (56.5%) | 0.76 |
| Malay (%) | 6 (20.7%) | 132 (26.9%) |  |
| Indian (%) | 5 (17.2%) | 81 (16.5%) |  |
| SER, Mean (SD) | ˗1.71 D (1.68) | 1.06 D (0.72) |  |

Abbreviations: SER, Spherical equivalent refraction; D, diopters; SD, standard deviation
