# Supplementary material for: In-utero epigenetic factors are associated with early-onset myopia in young children
Source: PLoS One. 2019 May 17;14(5):e0214791. doi: 10.1371/journal.pone.0214791 (PMC6524791; doi:10.1371/journal.pone.0214791)
Supplement: S2 Table — (DOCX) [file pone.0214791.s002.docx]

**Supplementary Table 2. Significant CpGs that are different between myopic cases and controls using continuous spherical equivalent refraction measures**

| CpG | Chr | Gene | Continuous SER | |
| --- | --- | --- | --- | --- |
|  |  |  | Estimate (95% CI)^a^ | P-value^b^ |
| cg03155767 | 4q31.3 | *FGB* | 0.23 (0.02, 0.44) | 3.22× 10^-2^ |
| cg14066632 | 12q23.2 | *ARL1* | 1.02 (0.73,1.31) | 2.17× 10^-11^ |
| cg17154092 | 18q23 | *PQLC1* | 0.30 (-0.10, 0.71) | 0.14 |
| cg21880079 | 8p23 | - | 0.49 (0.22, 0.73) | 3.00× 10^-4^ |
| cg26299044 | 17q21.2 | *KRT12* | 0.59 (0.34, 0.84) | 4.50× 10^-6^ |

Abbreviations: Chr, chromosome; CI, confidence interval; FDR, false discovery rate; SER, ; spherical equivalent refraction

^a^Regression coefficients (Estimate) are reported as percentage methylation change per 1 SD increase in SER measures.

^b^P-value was obtained from linear regression model of continuous SER measures and methylation at each CpG site, adjusted for sex, gestational age, ethnicity, bisulfite conversion batch and cellular proportions.
