# Supplementary material for: In-utero epigenetic factors are associated with early-onset myopia in young children
Source: PLoS One. 2019 May 17;14(5):e0214791. doi: 10.1371/journal.pone.0214791 (PMC6524791; doi:10.1371/journal.pone.0214791)
Supplement: S4 Table — (DOCX) [file pone.0214791.s004.docx]

**Supplementary Table 4. Significant CpGs that are different between myopic cases and controls at a 5% false discovery rate, adjusting for maternal smoking status**

| CpG | Chr | Gene | Estimate (95% CI)^a^ | P-value^b^ | FDR |
| --- | --- | --- | --- | --- | --- |
| cg03155767 | 4q31.3 | *FGB* | -2.39(-3.31,-1.48) | 4.36 × 10^-7^ | 0.023 |
| cg14066632 | 12q23.2 | *ARL1* | -3.53(-4.87,-2.19) | 3.13 × 10^-7^ | 0.023 |
| cg17154092 | 18q23 | *PQLC1* | -4.52(-6.3,-2.73) | 8.84 × 10^-7^ | 0.028 |
| cg21880079 | 8p23 | - | -3.06(-4.19,-1.92) | 1.79 × 10^-7^ | 0.023 |
| cg26299044 | 17q21.2 | *KRT12* | -2.84(-3.96,-1.72) | 8.66 × 10^-7^ | 0.028 |

Abbreviations: Chr, chromosome; CI, confidence interval; FDR, false discovery rate

^a^Regression coefficients (estimate) are reported as percentage methylation change in case group.

^b^P-value was obtained from linear regression model of myopia (case and control) and methylation at each CpG site, adjusted for sex, gestational age, ethnicity, bisulfite conversion batch, cellular proportions and maternal smoking status.
