# Supplementary material for: In-utero epigenetic factors are associated with early-onset myopia in young children
Source: PLoS One. 2019 May 17;14(5):e0214791. doi: 10.1371/journal.pone.0214791 (PMC6524791; doi:10.1371/journal.pone.0214791)
Supplement: S1 Fig — (DOCX) [file pone.0214791.s005.docx]

**Supplementary Figure 1. Q-Q plot for p-values from EWAS on umbilical cord methylation profiles from case-control comparisons**


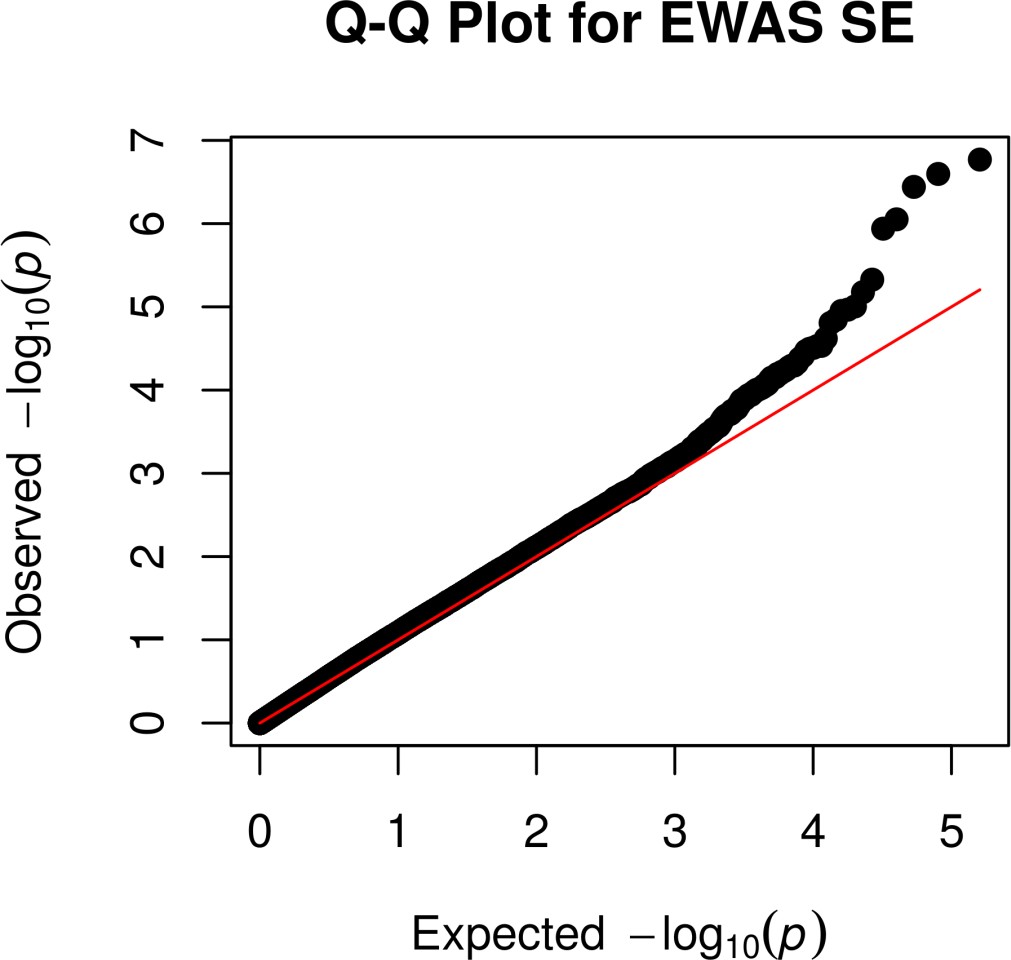


P-values were demonstrated by negative log10 transformation. The distribution of observed p-values for each CpG (dark dots) were compared to the distribution of expected p-values (red line). The distribution of observed P values generally followed the expected distribution for most of the CpGs, whereas, there were p-values higher than expected near genome-wide significance (FDR p-value < 0.05).
